# REVISITING THE EBOLA EPIDEMIC IN WEST AFRICA: THE ROLE OF EMOTIONAL DETERMINANTS IN PUBLIC RESPONSES

**DOI:** 10.21010/Ajidv17i2.3

**Published:** 2023-03-29

**Authors:** KOUSOULIS Antonis A, GRANT Imogen F., DUNCAN Joshua A, LARSON Heidi J.

**Affiliations:** 1 Vaccine Confidence Project, London School of Hygiene & Tropical Medicine, London, UK; 2 Mental Health Foundation, London, UK; 3 Mental Health Coalition, Freetown, Sierra Leone; 4 Dept. Health Metrics Sciences, University of Washington, Seattle, USA

**Keywords:** Ebola, epidemics, emotions, Sierra Leone, Liberia, Guinea

## Abstract

**Background:**

The 2014-2016 Ebola epidemic was largely restricted to the three nations of Guinea, Liberia and Sierra Leone, yet it tested the world’s ability to address a potential global pandemic. This study provides an in-depth examination of the role of emotions in the response to the outbreak and engagement with public health measures, and the contextual factors which influenced them.

**Methods:**

Historical research methods were utilised in the examination of primary and secondary sources. A multi-faceted SPEECH (Society and Politics, Economy, Epidemiology, Culture, Healthcare and Public Health) framework was developed to aid data synthesis and analysis.

**Results:**

The outbreak occurred in a region still reeling from years of civil war, where poverty was widespread and healthcare severely underfunded. Internationally, global health security had been politically neglected. After a slow start, the international response to the outbreak was strong, yet the lack of community engagement and inadequate consideration of local culture and traditional beliefs, fueled fear and hindered engagement with professionals and uptake of public health measures. Improved collaboration and communication with rural communities in the latter phases of the response was crucial in effectively addressing the outbreak.

**Conclusion:**

This study illustrates the importance of effective collaboration between international crisis responders, in-country public health practitioners and local communities in addressing public emotional responses to the Ebola outbreak. It highlights how community engagement and communications tactics can effectively be utilised to soothe and educate the public, abating counterproductive extreme emotional responses, and in turn improving uptake of public health measures.

## Introduction

The Ebola Virus Disease (EVD) outbreak in West Africa 2014 - 2016 was the largest ever recorded of its kind (Kaner and Schaack 2016). The outbreak tested the world’s ability to address a potential global pandemic (Thompson 2017), and put a spotlight on the vulnerability of societies to infectious disease threats spreading across national borders (Heymann 2015).

Since the virus’ first description almost four decades before (Breman 1976), all outbreaks had been rare, localised in rural areas and small (Kaner and Schaack 2016). This was an outbreak that occurred in a low resource setting, spreading to urban centres and killing more people than ever before, of a disease the standard treatment for which had not changed in the past 40 years (Del Rio 2014). This 2014 outbreak of “*an old virus in a new context*” in West Africa (World Health Organization 2015a), is thought to have exposed important deficiencies in the ability and capacity of the international scientific and public health community to respond to such epidemic emergencies (Kaner and Schaack 2016). It has also generated valuable learning on emotions as determinants of health outcomes and behaviour.

The aim of this case study is to examine the contextual factors which impacted upon public emotions in response to the 2014 Ebola outbreak across Guinea, Liberia and Sierra Leone, and the role that such emotions in turn played in the public’s risk perception and engagement with public health measures (Kousoulis 2022c). This examination of the public health implications of such emotional responses is particularly pertinent in light of the recent case of the highly virulent Marburg Virus Disease in Guinea (August 2021) of the same viral family as Ebola (World Health Organization 2021b, c), the Ebola virus outbreaks in the Democratic Republic of Congo and Guinea earlier this year, and the suspected case in Cote d’Ivoire in August (since, no evidence of EVD has been found in the patient)(World Health Organization 2021a, d), as well as the ongoing Covid-19 pandemic, with West Africa experiencing a resurgence of Covid-19 cases in recent months (World Health Organization 2021e). This case study seeks to generate findings valuable to the ongoing responses to infectious disease outbreaks and future epidemic preparedness in the region.

## Materials and Methods

This case study was conducted using historical research methods. Available primary sources were explored, including media and contemporary scientific journal articles, to gather evidence from the era. In addition, online search tools that indexed and documented available primary sources were explored. Selected secondary sources including historical books and peer-reviewed journal articles were also utilised.

There is an inherent challenge when studying responses to epidemics at the time when they emerged. This is particularly true when the infectious disease and nature of transmission were not entirely scientifically understood. Hence, complementing primary research with secondary sources or studies published retrospectively, helps bring perspective to the historical view.

### Rationale

Researching the impact of the role of emotions on individual or population responses to previous epidemics necessitates a historical lens. Research indicates that human emotions are made and learned in response to personal experiences and input from the surrounding world into feelings that are relevant to their context (Epstein 1994). Hence, emotions are influenced by numerous contextual factors, that is the contemporary social, economic, and cultural circumstances in which we exist (Kousoulis 2022c).

## Data Analysis

An interdisciplinary public health research framework incorporating a breadth of contextual factors (Slovic 2004) was required to conduct a comprehensive in-depth study of this historical outbreak. Thus, a multifaceted framework which focuses on a broad ecosystem of influences which impact upon emotions in relation to epidemic emergence and spread was developed. A rapid literature review was conducted based on a World Health Organization (WHO) systematic approach framework developed to inform health policy and systems (Vigh 2008). Key determinants were extracted and classified based firstly on the European Core Health Community Indicators (ECHIs), and subsequently refined through narrative summary (Koselleck 2006; Dicker 2011). Through further evidence synthesis of the key determinants identified, the SPEECH (Society and Politics, Economy, Epidemiology, Culture, Healthcare and Public Health) framework was developed (Kousoulis 2022b).

The SPEECH framework was utilised to systematise and contextualise evidence for this in-depth case study and enable commentary on how emotions play a role in public responses to infectious disease outbreaks (Kousoulis 2022b).

## Results

Epidemiology

The Ebola epidemic was largely restricted to the three nations of Guinea, Liberia, and Sierra Leone. A total of 28,656 cases were reported (and 11,325 deaths), of which only 36 were in nations outside of those three, with 20 of those in Nigeria and eight in neighbouring Mali (CDC, 2016). The epidemic spread from an initial case of an 18-month old boy from a village of southern Guinea, who was infected by contact with bats in December 2013 (Baize 2014). It reached the nation’s capital in March 2014 and the outbreak was officially declared by the WHO on March 23^rd^, with 49 confirmed cases and 29 deaths (World Health Organization 2015a). It subsequently grew exponentially between June and September in Guinea, Liberia and Sierra Leone, where the national case number was doubling every 16 to 30 days (Ebola Response Team 2014).

A significant factor for the emergence of the epidemic appears to have been the deforestation of the Forest Region of the Gueckedou District in Guinea. Foreign mining and timber operations led to loss of more than 80% of forest, bringing wild animals and bats (the natural reservoir of Ebola virus) closer to human settlements resulting in the contact of the index case with insectivorous bats in his backyard (World Health Organization 2015a).

The larger geographical spread and case numbers of this EVD outbreak has been attributed to several factors (World Health Organization 2015a, CDC, 2016):

i) This was the first EVD outbreak to spread in crowded urban areas, also with low resources and poor public health infrastructure (this also meant early detection and relevant clinician expertise were poor).
ii) Local social determinants, including damaged infrastructure and lack of education in the three nations that had only recently emerged from years of civil war.
iii) High mobility of people across porous country borders.
iv) Conflict between international infection control practices and local cultural and traditional practices (including unsafe burials and contact with bodies of the deceased).
v) Weak local surveillance systems.

### Society and Politics

Criticism of the WHO’s lack of decisive, early response to the outbreak is widespread (Honigsbaum 2017). It’s failure to strengthen its local emergency support and outbreak control until the epidemic was already “out of control” has been highlighted (Médecins Sans Frontières 20 June 2014). The reasons that have been provided include underfunding for tropical emerging infectious diseases (Lawrence 2015), gaps in the leadership and governance issues in the organisation’s headquarters (Moon et al. 2015), political relationships driving an unwillingness to declare an emergency as it would be detrimental to the local economy, trading and international business in relation to the affected countries (25 March 2014), and a general underestimation of the capacity of the virus to cause such widespread damage (Honigsbaum 2017).

These shortcomings were underpinned by the wider global context of health security at the time. By the 2010s the global health security strategy had suffered political neglect by countries internationally – and subsequently been downgraded within the WHO – well before the outbreak of Ebola in West Africa (Press 2014; Heymann 2015). Although several nations stepped in once the outbreak had escalated into a crisis, the scale of the outbreak and surge of need is illustrative of the wider lack of political commitment to global health security which had been noted before the outbreak (Fidler 2015).

In the face of the delayed international response, local leadership in Sierra Leone, Guinea and Liberia played a critical part in addressing the crisis. Whilst the first phase of the epidemic was hampered by weak decision-making, the existing hierarchical leadership model was subsequently replaced by a distributed approach. This more equitable model enabled meaningful engagement with local stakeholders (Nyenswah et al 2016). Crucially, it empowered local, traditional leaders to effectively combat misinformation and work collaboratively with public health professionals in the latter phases of the epidemic (World Health Organization 2015b). Understanding communities’ traditional beliefs and customs, and securing endorsement from local leaders, was critical for building trust between local communities and public health professionals.

### Economy

The region was only starting to recover from years of civil war, and poverty, damaged infrastructure, and lack of education were widespread (World Health Organization 2015a). War and famine had taken a huge toll on the population and are both well recorded determinants of epidemic emergence (Morens 2008, Kousoulis 2022b). Poverty and periods of recession are also key drivers of morbidity, mortality and distribution of infectious disease ( Morens 2008; Suhrcke 2011; Kousoulis 2022b). In 2013, prior to the Ebola outbreak, Liberia and Sierra Leone were countries with some of the highest poverty headcount percentages based on the UN Development Programme (UNDP) Multidimensional Poverty Index, ranked at 85% and 77% respectively (Malik 2013). Guinea, Sierra Leone and Liberia were also ranked in the lowest category of the 2013 UNDP Human Development Index (Malik 2013). The subsequent Ebola outbreak and response contributed to further economic decline and exacerbated poverty and widespread hunger, with borders and markets forced to close (Shultz 2015).

Prior to the epidemic, healthcare in all three predominantly affected nations was severely underfunded. Data from 2012 show that the Guinean government spent $9 per person per year on healthcare, the Sierra Leonean $16, and the Liberian $20. All far below the WHO recommendation of a minimum of $86, necessary to provide essential health services (Wright 2015).Health systems in all three countries lacked the funding and capacity to expand care to all at the point of need. The epidemic stretched them further and even following the end of the emergency, many survivors had to wait months to undergo surgery for cataract and others were re-traumatised as they were being re-tested for Ebola at the point of admission (Moses 2017).

### Healthcare and Public Health

Following a slow start, with the realisation of the limited local resources, came a strong international response to the outbreak. By the spring of 2015, there were 176 organisations operating emergency programmes in Guinea, Liberia, and Sierra Leone, reaching a point where the total number of Ebola Treatment Unit beds exceeded the number of reported patients and enough safe burial teams were also in place (Kaner and Schaack 2016, Ki-moon 2015). However, the distribution of these resources was not adapted to the geographic spread of Ebola and the international community did not start by effectively addressing the continued fear and suspicion of Ebola treatment hospitals and burial teams in local communities. This resulted in many patients still going without treatment or safe burials which led to new infections (Ki-moon 2015).

A public health measure that exacerbated local suspicion was the enforcement of mass quarantines. All three affected nations implemented quarantines in large forest areas around their shared borders (Eba 2014). The Liberian government went further to implement a 10-day quarantine in August 2014, enforced by soldiers in the country’s largest slum, West Point, housing approximately 75,000 residents as reported in a post by Hildebrandt on August 25, 2014 (Hildebrandt 2014). The Sierra Leonean government implemented a three-day nationwide mass quarantine as reported in Nossiter in September 2014 in an effort to find patients hiding across the country (Nossiter 2014). No effort had yet been made to engage local communities when these measures were introduced, resulting in exacerbated uncertainty, anxiety and fear in communities, with Human Right Watch reporting cries that rights to liberty and security were being violated (Eba 2014, Watch 2014), and an increase in the incidence of people exposed to Ebola hiding from healthcare services as reported by AOL (Roy-Macauley 2014). The violent enforcement of these measures using military troops, in an environment of existing mistrust, further alienated the very people whom the response sought to engage (Eba 2014).

The outbreak also had a significant impact on healthcare human resource. Not only did public attitudes towards healthcare workers vary hugely during the epidemic, but healthcare personnel called to care for people with Ebola were among those at highest risk for contracting the infection. During the epidemic, Liberia lost 8% of its doctors, nurses, and midwives to EVD (Evans 2015). In addition to these devastating effects on the workforce in Liberia, Sierra Leone and Guinea, the epidemic had a severe impact on the provision of healthcare services and caused setbacks in the treatment and control of tuberculosis, malaria, measles, and HIV in these countries (Parpia 2016). The risks to health workers’ health security created a vicious cycle, whereby Ebola-infected West Africans had to accept that health care was not always safe, not always effective, and not always accessible, meaning their own health security was constantly at risk (Heymann 2015).

Vaccine trials began swiftly and included two studies in Ghana. However, they soon illustrated the role of rumours in the spread of fear, uncertainty and distrust. The resultant controversy and dispute between health authorities, political actors and the public, escalated to the point of trials being suspended (Kummervold 2017).

The main concerns, as communicated by claims made in the media, were that the vaccine trials would cause an Ebola outbreak in Ghana, and the incentives offered to participants were compensation for the trials’ risks which included that the vaccine might give study participants Ebola (Kummervold 2017). This exacerbated public mistrust of authorities in Ghana, a setting where knowledge of Ebola was low, and misconceptions were persistent. For example, fear locally was intensified by the fact that huge colonies of fruit bats (which were thought to be a host for EVD) existed in the country and restrictions were being imposed on the consumption of bushmeat, which was a local delicacy (Thompson 2017).

### Culture

The direct transmission of the disease brought challenges to local communities during the Ebola epidemic. Several funeral and burial practices in West Africa are considered exceptionally high risk from a public health perspective. This includes mourners bathing in or anointing others with rinse water from the washing of corpses, especially in Liberia and Sierra Leone, or people sleeping near the bodies of the deceased for several nights believing that this practice enables the transfer of powers (World Health Organization 2015a).

In the first year of the epidemic, as many as 60% of cases in Guinea were linked to these traditional practices (Kaner and Schaack 2016). However, it was not just burial practices that presented a challenge. Simple everyday actions, such as caring for loved ones in the family home, parents holding their children who were ill, or nurses compassionately touching their patients frequently led to transmission of the virus. The instructions from public health professionals to reduce human contact brought alarm and hopelessness (Aylward 2014).

Early media reports from Guinea and Liberia heavily framed Ebola as a highly infectious condition causing terrifying symptoms (Honigsbaum 2017). These spread further afield and induced panic in other

African nations about the possibility of transmission through air travel (Thompson 2017, Bogoch 2015). However, communities in the three affected nations were sceptical of this media-driven image of Ebola and conspiracies emerged (Honigsbaum 2017). In Liberia, especially, media communications and the quarantine measures reinforced rumours that the outbreak was a measure taken by the government to manage population growth and attract international funding (Thompson 2017). In Sierra Leone, opposition parties suggested that the political party in power, the All People’s Congress (APC), was trying to coerce the opposition population (Duncan 2021). The real motivations of the foreign medical teams were questioned in all three countries and this social resistance was further aggravated when bans on traditional burials and limits on social interactions were introduced (Honigsbaum 2017).

The misunderstandings, suspicions, and the deadly nature of the condition gradually cultivated considerable stigma against people showing symptoms, those perceived as exposed to the virus, and healthcare workers (Moses 2017). Whole groups of people were shunned due to a toxic mix of scientific ignorance and fear-based communications (Gonsalves 2014). This stigmatisation extended globally to encompass, in the eyes of international communities, entire countries with confirmed cases of EBV and, in fact, the whole of the African continent (Shultz 2015). The media hype, the generic use of “Africa” and “Ebola” in the coverage of the epidemic, and the persistent lack of engagement with the geographical and cultural differences between the various African nations, led to a wider ecology of fear impacting perceptions around the world.

**Figure 1:**
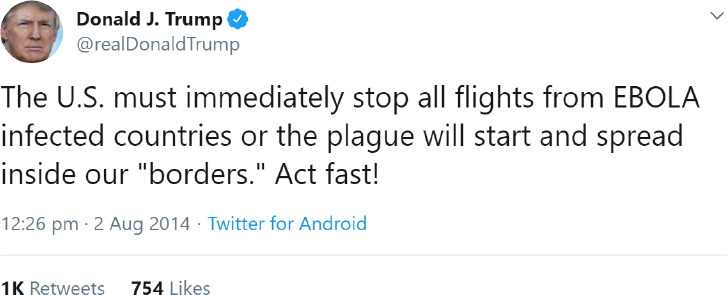
Post on Twitter by Donald Trump demanding air traffic control to stop the spread of Ebola

**Figure 2:**
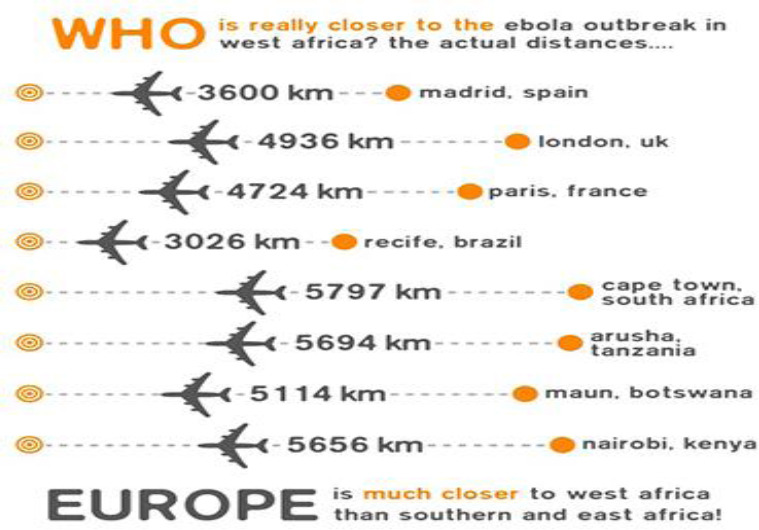
“Adventure Life” specialist travel agent tried to explain how low the risk of exposure to Africa is in many African nations advocating for more holiday in southern and east Africa.

A concern that the Ebola crisis might trigger large scale migration, which would in turn enable further spreading of the virus, emerged. Despite evidence from previous health crises showing that travel bans are not always effective and their unintended consequences can sometimes mean they are more harmful than beneficial (Edelstein 2014), restrictions were imposed. Several neighbouring countries closed their borders, remote countries, such as Australia imposed temporary visa restrictions, and several airlines reduced flights to the affected countries, or, in some cases, to the whole of the African continent (Koser 2015). Influential figures, including the then future President of the USA, Donald J. Trump, took to social media to demand air traffic control (Figure 1), only adding to the global panic reactions. This was particularly harmful to the tourist economy, which saw a universal drop in visitors despite a complete lack of risk of exposure to Ebola (including an over 50% loss in safari bookings for the year 2015). Figure2 shows a specialist travel agent’s attempt to reassure tourists holidaying in various African nations, using epidemiological evidence and images to help clarify the risks(Life 2015).

## Discussion

The Ebola outbreak has been repeatedly referred to as one in which fear and panic spread quicker than the infection, and is thus an insightful case study for increasing understanding of the role of emotional determinants in public health (Aylward 2014). In the face of early and persistent denial that Ebola was real within the general population of the affected nations, partly due to overlapping symptomology with other endemic diseases and socio-political factors, health messages issued to the public repeatedly emphasised the deadly and untreatable nature of the disease. Whilst intended to promote protective behaviours in the public, these messages had the opposite effect, fueling hopelessness and despair (World Health Organization 2015a). A systematic review conducted in conjunction with this research (currently in publication process) found that cases in which fear and panic (about both the disease itself and the response) were evoked during the Ebola epidemic, did not lead to public uptake of protective measures (such as staying at home or following personal hygiene methods) (Kousoulis 2022a). In fact, fear and panic proved to be counterproductive and a barrier to taking appropriate action (Yamanis 2016, Desclaux 2017).

Furthermore, the fear and despair elicited by the early response to the outbreak fostered stigma against those who currently had or had survived the disease, health workers in treatment centers, and those who had hidden an infected family member and unintentionally precipitated an outbreak within the community. To combat the conflict within communities borne of the high levels of stigma and fear, communication tactics were utilised to help combat misconceptions. For example, survival stories broadcast on local and national radio were effectively utilised to shift perceptions, framing Ebola survivors as local heroes who had emerged triumphant from “the valley of the shadow of death” (Duncan 2021). Such positive, hopeful media messaging also effectively promoted public health guidance and engagement with public health professionals, e.g., by broadcasting success stories of how timely treatment improves likelihood of recovery and survival. This supports the growing body of evidence on the effectiveness of evoking pleasant emotions, such as hope and empathy, as motivators for public health intervention uptake, and this is an area of emotional determinants which warrants further investigation (Kousoulis 2022a). The Ebola outbreak and response highlights both the highs and the lows of the ability of mass media communications and social media to disseminate messaging and influence public opinion. From the early media reports stoking panic across the African continent, and the world, to later successful efforts to spread focused messaging to the public through uplifting stories of survival resulting in reduced stigma and increased adherence to public health guidance.

Compassion seems to have inadvertently played a role in enabling the spread of the Ebola Virus. Although initially contradictory to emerging evidence that pleasant emotions such as empathy, hope and compassion, tend to have a motivating role in public engagement with protective public health measures during an outbreak; compassion in this case is meant as a cultural trait, rather than an emotion elicited in response to public health communications or another trigger. In its year-one report on the epidemic, the WHO noted that Ebola Virus seemed to have spread through networks bonded together by a culture encouraging compassionate care for the ill and ceremonial care for the deceased bodies. There also existed records of doctors most likely getting infected because they rushed to aid patients who collapsed in waiting rooms or outside hospitals with no hygienic protection (World Health Organization 2015a). The despair and hopelessness brought about by public health professional’s instructions to reduce human contact, particularly when caring for the ill, further illustrates how a lack of understanding of entrenched behaviours and cultural traits hindered an effective early response to the outbreak (Aylward 2014).

The threat of the virus was overwhelming to local communities, and the pervasive fear which had a significant impact on public behaviours was compounded by healthcare providers’ lack of communication with families in the early stages of the epidemic (Aylward 2014). When patients were taken to treatment or transit centres, anxious families often received little information about the patient’s condition, outcome, or even the place of burial (World Health Organization 2015a). In other cases, such as in Monrovia, Liberia, when Ebola was identified as the causative agent of the epidemic, several members of staff left the hospital in fear (Moses 2017). For the public in West Africa this meant that if hospitals and international aid were not able to offer any treatments or therapies, many families preferred to care for their loved ones at home, or in some cases even hiding patients in their homes. In their view, if death was almost inevitable and the hospitals were not transparent, they should letthis happen as comfortably as possible at home, among familiar faces (Nouvet 2021). Subsequently – but perhaps too late for thousands of families – several experts noted that in the case of the Ebola crisis, the well-studied issue that control efforts must work within the culture, not against it, emerged (Hussen, 2020). When technical interventions cross purposes with entrenched cultural practices, culture is very influential in either shaping behaviours or the trust in the system (World Health Organization 2015a).

It is no coincidence that the response to the outbreak became significantly more effective once international efforts became more focused and professional co-operation with local communities increased. The failure to adopt community engagement methods and take emotions into account was initially neglected, yet eventually proved effective in winning the trust of rural populations (Honigsbaum 2017). Anthropological observations showed that the treatment of Ebola had strongly focused on the biomedical aspects alone and disregarded parameters such as community, society, and culture. Consequently, the emotions – expressed as fears and concerns – and traditional beliefs of the members of local communities were taken more seriously (Allgaier 2015, Fassassi 2014). This latter phase engagement with local, traditional leaders and efforts to understand local communities’ traditional beliefs and customs were crucial in building trust between health professionals and the public. This enabled strategies such as training staff working in local communities to teach basic self-care practices (e.g., breathing techniques), and supporting communities to grieve without traditional burial and funeral practices, to help address the pervasive grief and fear.

Although this case study largely examines the role of emotional determinants which emerged at a local level, fear at the global level also impacted policy makers and triggered public health measures, such as international travel restrictions, with repercussions for local populations and emotions. People feared not only the disease, but the inability to feed their family due to market and border closures restricting the movement of goods. The knock-on effects of the early international emotional response, one of fear and panic, stoked by media reports, thus had their own role in dictating the emotions and livelihood of the populations of the directly affected nations.

The Ebola crisis also exposed inequity in global development that ultimately shaped a societal vulnerability and a lack of empowerment for communities to respond effectively and rationally. Emotions became more prominent in the outbreak response with local communities and organisations insufficiently empowered with education, adequate resources, and strong health systems so as to better diagnose and respond to their own crises (Chen 2015). Thus, they were left dependent on a top-down global response, not effectively linked to local action.

## Conclusion

This in-depth case study examines the complex and interrelated social, economic, emotional, political and cultural factors that contributed in tandem to the emergence and spread of the Ebola epidemic in West Africa, as well as the public’s responses and behaviours. Hope, health education, and collaboration between local communities and healthcare practitioners were decisive factors in tackling the epidemic, compared to the earlier stages when fear, hopelessness, panic and desperation were dominant (Aylward 2014).

A key learning from the epidemic was the cruciality of partnership between those with international crisis response expertise and national public health personnel with cultural knowledge and the ability to engage local communities and leaders, in turning the tide on the epidemic. The symbiosis borne of a collaborative approach enabled the emotional responses of the population, from early disbelief and indifference followed by debilitating fear and panic, to be addressed through communication and community engagement on the ground, and in turn motivating adherence to and cooperation with public health measures.

The 2014 Ebola epidemic in West Africa triggered a contagion of fear internationally, in a way that has not been seen in recent history before the COVID-19 pandemic. Ebola has not been the last new and lethal pathogen to emerge. The COVID-19 pandemic has clearly shown that in today’s globalising world, we have a new context for infectious pandemics – larger human populations, unprecedented volume of transnational movement, rapid travel, and growing global inequalities in economics and health. What made Ebola different from COVID-19 and the many other epidemics was the fear of contagion that the lethal disease had precipitated among the public of high-income countries when the threat was predominant in some low-income nations. As such, it generated learning for next time that public health organisations should not wait for high-income countries to feel threatened to accordingly redirect global political priorities.

### Conflict of Interest

HL reports grants from GSK for a study on maternal vaccine acceptance and grants from Merck for research on health-care provider vaccine hesitancy and is a member of the Merck vaccine confidence advisory board.

**Abbreviations:**
APC: All People’s Congress;
Covid-19: Coronavirus Disease 2019;
EVD: Ebola Virus Disease;
UNDP: United Nations Development Programme**;**
WHO: World Health Organization.
